# Spontaneous rectus sheath hematoma as a differential diagnosis for localized abdominal swelling in chronic liver disease: A rare case report

**DOI:** 10.1002/ccr3.9474

**Published:** 2024-10-20

**Authors:** Shritik Devkota, Samiksha Lamichhane, Saurabh Baghi, Suraj K.C., Harsha Bhola

**Affiliations:** ^1^ Department of Radiodiagnosis & Imaging Anil Baghi Hospital Punjab India; ^2^ Department of Radiodiagnosis & Imaging B. P. Koirala Institute of Health Sciences Dharan Nepal; ^3^ Department of Cardiology Anil Baghi Hospital Punjab India; ^4^ Department of General Surgery B. P. Koirala Institute of Health Sciences Dharan Nepal; ^5^ Department of General Surgery Anil Baghi Hospital Punjab India

**Keywords:** chronic liver disease, rectus sheath hematoma

## Abstract

**Key Clinical Message:**

Spontaneous rectus sheath hematoma is a rare complication in chronic liver disease patients. Early clinical suspicion with prompt radiological evaluation is crucial for accurate diagnosis and timely management.

**Abstract:**

Spontaneous rectus sheath hematoma can present as an acute abdomen in the emergency department. The rupture of the upper and lower epigastric arteries and their branches is the main cause of hematoma formation. Hepatic dysfunction can affect the clotting process, increasing the risk of hematoma development. Computed tomography is the preferred diagnostic tool. Most hematomas can be managed conservatively, with only a few requiring minimal intervention or surgical management. We report an uncommon instance of spontaneous rectus sheath hematoma in a patient with chronic liver disease presenting with painful abdominal distention, mimicking a hernia and initially posing a diagnostic challenge. The rectus sheath hematoma was definitively diagnosed through clinical and radiological evaluation and subsequently evacuated with successful outcomes.

## 1 INTRODUCTION

Spontaneous rectus sheath hematoma is a rare entity. Rupture of epigastric vessels or tearing of the rectus muscle leads to the accumulation of blood in the sheath, which is usually benign and termed rectus sheath hematoma.^1^, ^2^ In the evaluation of patients with chronic liver disease presenting with a novel, progressively enlarging, nonpulsatile abdominal mass accompanied by localized pain, spontaneous rectus sheath hematoma should be included in the differential diagnosis despite the absence of a documented traumatic event. We report an uncommon presentation of spontaneous rectus sheath hematoma manifesting as painful abdominal swelling. The clinical picture was atypical, making initial diagnosis a challenge. However, prompt clinical evaluation followed by ultrasonography and computed tomography facilitated the accurate identification of rectus sheath hematoma, enabling successful management.

## 2 CASE PRESENTATION

### 2.1 Clinical history and clinical examination

A 56‐year‐old male with a known history of alcoholic liver disease and repeated hospital admissions presented to the emergency department with complaints of gradually increasing painful abdominal swelling. The pain was described as burning in nature and severe enough to restrict his daily activities, exacerbated by movement. He had difficulty breathing and lying down. There were no overlying skin changes. He gave a vague history of an increase in the size of the swelling while rising from the bed. There was no history of any trauma, chronic cough, or bleeding from any orifices. He was not under any anticoagulation drugs or aspirin. Additionally, there was no family history of bleeding disorders or prior history of bleeding disorders in the patient. There was no history of fever, discharge from the swelling, abdominal distension, or changes in bowel and bladder habits. The patient had a significant history of alcohol consumption, drinking approximately 1–2 L of locally made alcohol regularly for the last 10 years. His last intake of alcohol was 4 days before the presentation. The patient's past medical history was significant only for alcohol‐related liver disease. No prior surgeries or significant medical events were documented. The family history was negative for liver disease and malignancy.

At presentation, he had a normal blood pressure of 100/60 mmHg, a heart rate of 125 beats per minute, and a respiratory rate of 18 breaths per minute. The patient was afebrile with a temperature of 36.3°C. He was conscious, cooperative, and well‐oriented to time, place, and person. A general physical examination revealed icterus. There were no signs of hepatic encephalopathy or peripheral stigmata of chronic liver disease.

**FIGURE 1:**
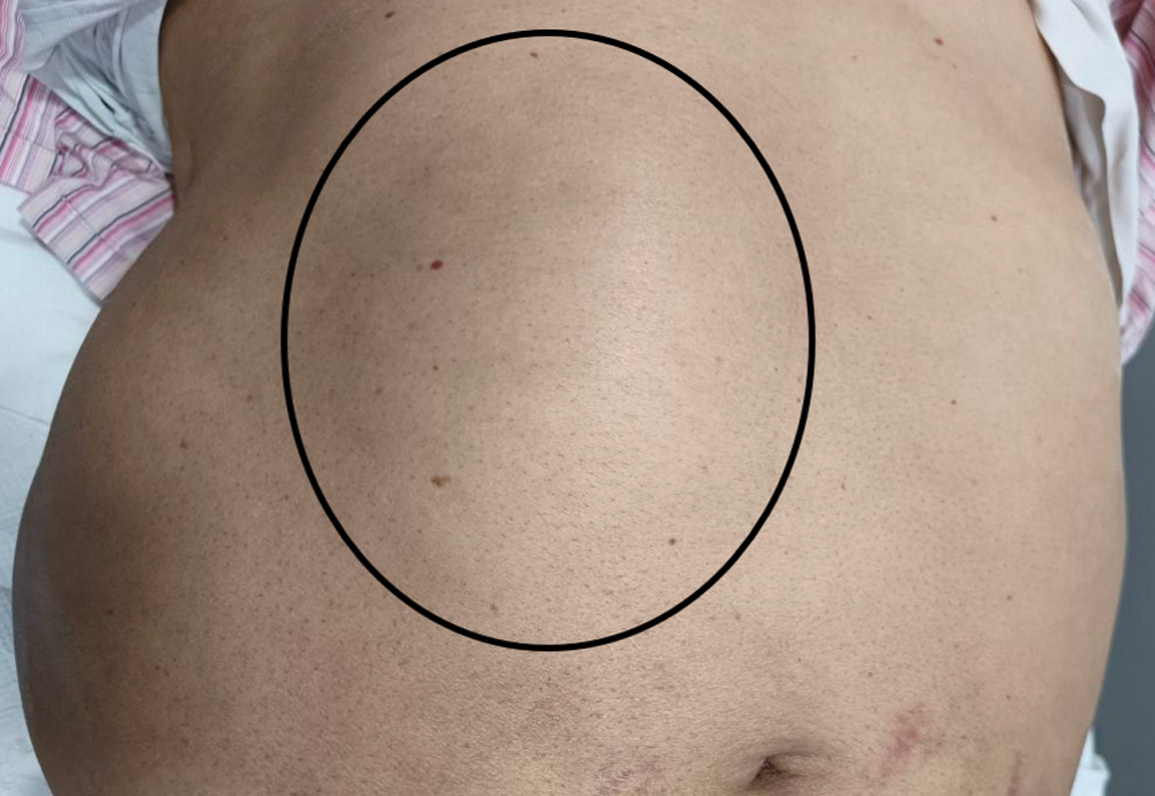
Clinical picture showing focal swelling (black circle) in right upper quadrant and epigastrium.

On systemic examination, the abdomen was distended and soft; however, there was a bulge over the right upper quadrant that was irreducible, with no cough impulse. The swelling extended from the right subcostal margin up to the right lumbar region (Figure 1). There were no signs of ascites. There was no hepatosplenomegaly on clinical palpation.

### 2.2 Investigations

Laboratory evaluation revealed a concerning clinical picture. Macrocytic normochromic anemia (Hb 7.0 g/L) with associated thrombocytopenia (platelets 37 × 10^9^/L) was identified. The coagulation profile demonstrated significantly prolonged INR (3.0), PT (31 s), and PTT (35.2 s), suggesting a heightened bleeding risk. Liver function tests (LFTs) disclosed low total protein (5.1 g/dL) with concomitant hypoalbuminemia (1.5 g/dL). Mildly elevated bilirubin (2.5 mg/dL) predominantly comprised indirect bilirubin (0.9 mg/dL). LFTs also showed a prominent aspartate aminotransferase (AST) elevation (200 U/L), while alanine aminotransferase (ALT) remained mildly elevated (40 U/L) with an AST/ALT ratio of 5, suggestive of alcoholic hepatitis. Elevated LDH (490 U/L) and GGT (66 U/L) further supported the possibility of tissue damage. Notably, viral markers for hepatitis B and C were negative. These findings strongly suggested significant hepatocellular dysfunction, potentially consistent with alcohol‐related chronic liver disease.

**FIGURE 2:**
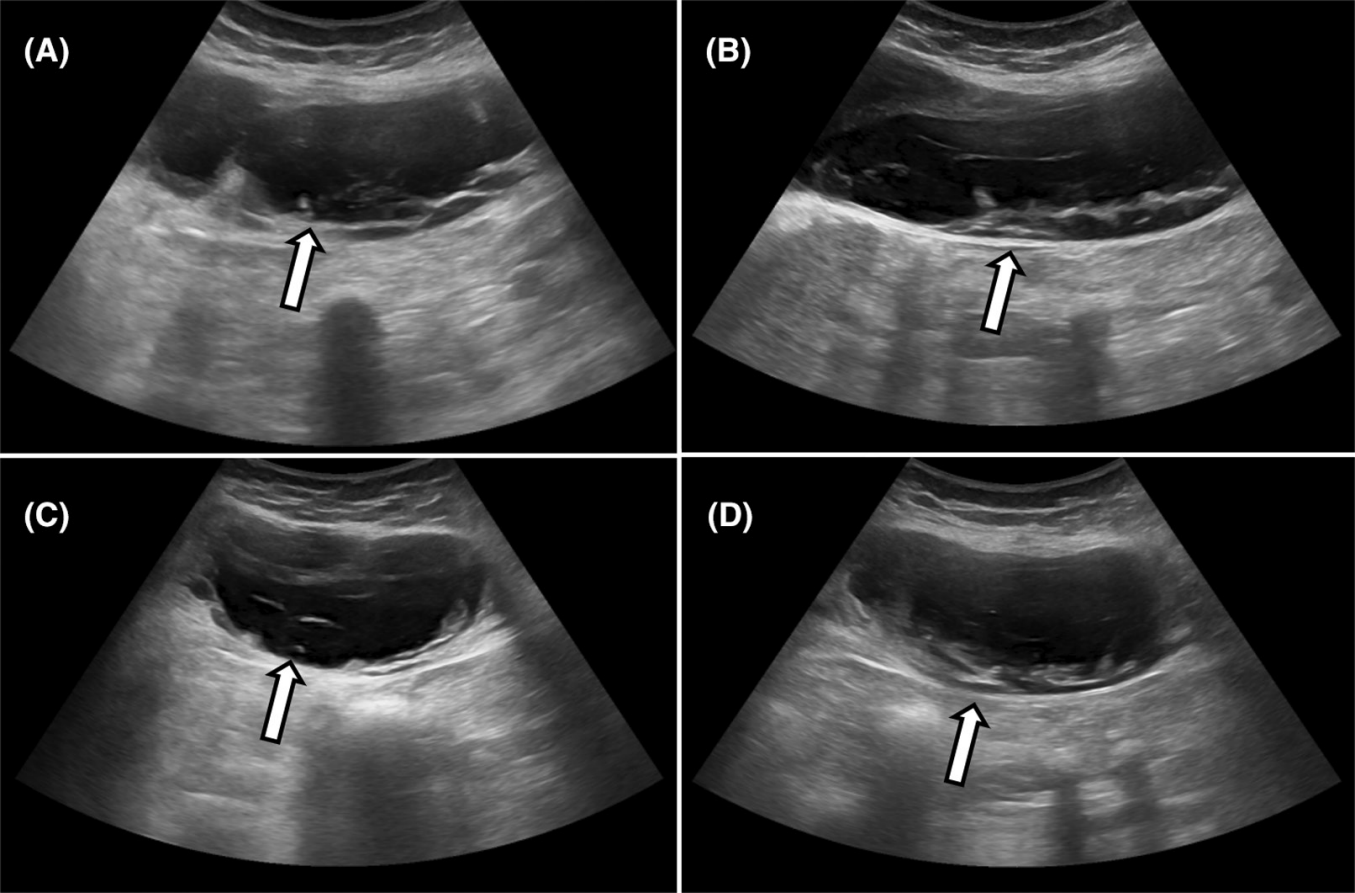
Ultrasonography (A, B, C, D) images showing heterogeneously anechoic lesion (white arrows) replacing right rectus abdominis muscle with few septations and internal echoes.

Ultrasonography of the local region (Figure 2) illustrated an ill‐defined heterogeneously anechoic lesion with septations and internal echoes in the intermuscular compartment within the right rectus abdominis muscle with no internal vascularity. No underlying defect was noted. No thrombus was noted in the veins. Overlying subcutaneous edema was noted.

**FIGURE 3:**
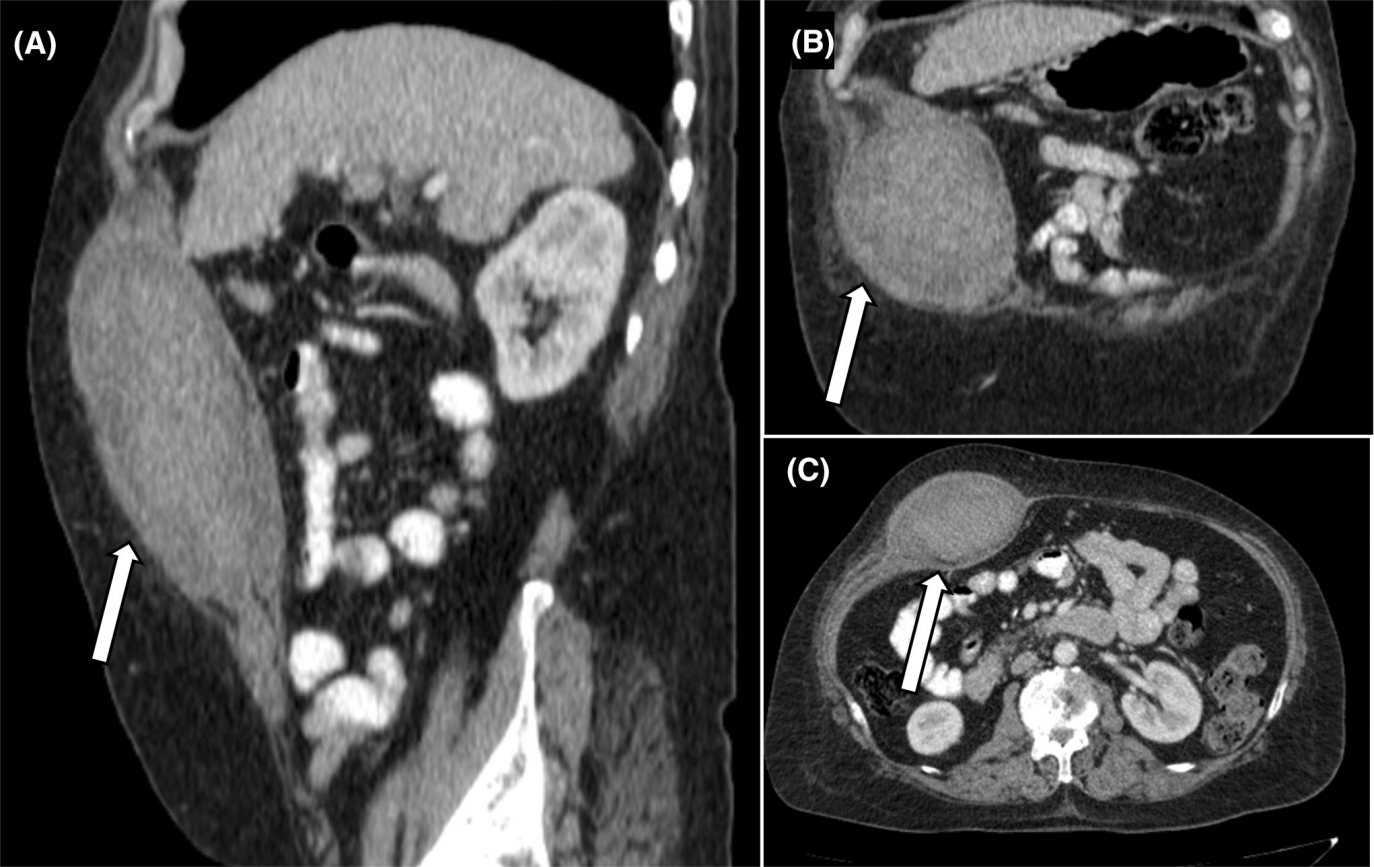
Sagittal reformatted (A), coronal reformatted (B), and axial (C) images of CT abdomen showing heterogeneously hyperdense lesion (white arrows) in right rectus abdominis muscle and nodular shrunken liver in (A) and (B).

The computer tomography angiogram (CTA) for the major abdominal arteries and branches was normal without any vascular cause for hematoma. A heterogeneously hyperdense area of size approx. 6 cm × 11.2 cm × 15 cm (anteroposterior × transverse × craniocaudal dimensions) (average attenuation ~64 HU) was seen involving the right rectus abdominis muscle, thus confirming the diagnosis of rectus sheath hematoma (Figure 3).

### 2.3 Treatment

The patient was managed with the transfusion of two pints of fresh whole blood and two pints of fresh frozen plasma. Intravenous tranexamic acid 1 g was given every 8 h for 7 days, with transfusions repeated over 3 separate days during hospitalization, as well as vitamin K 10 mg titrated against his serial INR measurements. He was given adequate pain relief with paracetamol and tramadol. Given the large size of the hematoma, surgical evacuation of the hematoma was done and the procedure was uneventful. A compression bandage was applied, and an abdominal binder was also prescribed. He was kept on a high‐protein diet and admitted for 10 days. His pain was subsiding, so he was planned for discharge with adequate antibiotic prophylaxis (Co‐amoxyclav and metronidazole).

### 2.4 Follow‐up

At his 1‐week follow‐up, he reported feeling well. To support his long‐term recovery, he was advised to abstain from alcohol and participate in a local social motivational and support group.

## 3 DISCUSSION

Spontaneous rectus sheath hematoma accounts for approximately 2% of patients who present to the emergency department with acute abdominal pain, with an incidence of around 1.2–1.5 cases per year in patients referred for radiological evaluation of abdominal pain.^2^ Risk factors for hematoma include female gender, advanced age, hypertension, atherosclerosis, hematologic diseases, collagen vascular disorders, degenerative muscle diseases, intra‐abdominal injections, paracentesis, peritoneal catheter insertion, pregnancy, obesity, blunt trauma, abdominal surgery, excessive and uncontrolled exercise, and increased abdominal pressure from coughing or sneezing.^3^

The cause for hematoma formation is the rupture of the upper and lower epigastric arteries and their branches or the rupture of the rectus muscles themselves during contraction.^4^ Liver cirrhosis and other chronic liver diseases rank as the 14th most common cause of death globally, significantly contributing to mortalities and disability‐adjusted life years (DALYs).^5^, ^6^ Chronic liver disease is a spectrum of advanced stages of various liver diseases such as hepatitis B and C infections, nonalcoholic fatty liver disease, alcohol consumption, autoimmune disorders, and more.^6^, ^7^, ^8^

Among the many functions of the liver, the production of proteins essential for coagulation is crucial. Coagulation factors, anticoagulants, proteins involved in fibrinolysis, and the platelet production regulator thrombopoietin are produced by the liver.^8^, ^9^ Hepatic dysfunction affects the clotting process due to these reasons. The regulation of clotting and thrombolysis is impaired in patients with cirrhosis. Natural anticoagulants, including antithrombin, inactivate thrombin and the active forms of factors X, IX, XI, and XII are also affected in liver cirrhosis.^8^ Hypersplenism secondary to portal hypertension and shunting of blood into the peripheral circulation induces a consumptive coagulopathy, further worsening thrombocytopenia and increasing the risk of bleeding.^10^, ^11^

Diagnosing rectus sheath hematoma can be challenging due to its nonspecific symptoms. However, certain clinical features can raise a red flag and prompt further investigation. The most common presentation is sudden, abdominal pain that worsens with movement or straining, often localized to the lower abdomen where the hematoma resides. A localized periumbilical or flank bruise may be present. A tender or non‐tender firm nonpulsatile mass may be palpable. In severe cases, signs of blood loss like weakness, dizziness, or lightheadedness might arise, particularly if bleeding is significant or the hematoma ruptures. Additionally, some patients may experience fever and chills, especially if an infection develops within the hematoma.^1^, ^2^, ^12^, ^13^, ^14^, ^15^

Rectus sheath hematoma can mimic other abdominal conditions, making diagnosis challenging. While clinical evaluation is important, radiology is essential for confirmation. Ultrasound, though readily available, has limitations in sensitivity and specificity. CT scan emerges as the gold standard tool for diagnosing rectus sheath hematoma with very high sensitivity.^12^, ^15^ This accuracy allows for not only confirmation but also precise localization, size determination, and differentiation from other abdominal wall issues. Additionally, CT can detect potential complications like bleeding. Early and accurate diagnosis with CT facilitates targeted management, avoids unnecessary interventions for mistaken conditions, and allows for timely detection of complications, ultimately improving patient outcomes. For patients where ionizing radiation exposure is a significant concern, such as pregnant women, magnetic resonance imaging (MRI) can be a safe alternative modality for diagnosis.^15^

Berna et al.^16^ proposed a three‐tiered CT‐based classification system to categorize rectus sheath hematoma severity, aiding in treatment decisions. This system stratifies rectus sheath hematoma based on the location and extent of bleeding visualized on CT scans. The least severe, Type I, presents with isolated bleeding confined within the rectus abdominis muscle, appearing enlarged and oval on scans. Type II, in addition to intramuscular bleed shows bleeding tracking between the muscle and a deeper layer of tissue, potentially affecting one or both sides. The most severe, type III, involves bleeding beyond the muscle, potentially into the abdominal cavity and associated with a significant drop in red blood cell count. This classification system facilitates tailored treatment based on the severity of the rectus sheath hematoma.

Management of rectus sheath hematoma is dictated by severity. Conservative management remains the mainstay therapy for hemodynamically stable patients with non‐expanding hematoma. These hematomas often exhibit a self‐limiting course, resolving spontaneously within weeks with close clinical monitoring and a treatment regimen that includes analgesia for pain management, ice compression for swelling, and activity restriction to minimize further risk of bleeding.^12^, ^13^, ^15^, ^17^, ^18^ For patients experiencing hemodynamic compromise, aggressive fluid resuscitation may be necessary to achieve stability. Blood transfusion is considered for those with significant blood loss or a precipitous decline in hemoglobin concentration.

In the setting of persistent hemodynamic instability despite fluid resuscitation, ruptured rectus sheath hematoma, or overt signs of infection, a more definitive operative intervention becomes mandatory.^12^, ^13^, ^15^ This encompass definitive hematoma evacuation, meticulous bleeding vessel ligation, and closed‐system drainage for persistent fluid collections. Percutaneous transcatheter arterial embolization (TAE) of the epigastric artery represents a minimally invasive approach for achieving hemostasis.^13^, ^19^ If TAE proves infeasible, surgical exploration with definitive hemostasis remains the ultimate resort.

## 4 CONCLUSION

In essence, this case report underscores the critical importance of considering spontaneous rectus sheath hematoma in the differential diagnosis for patients with chronic liver disease presenting with acute localized abdominal swelling. Prompt clinical suspicion followed by comprehensive evaluation, including both clinical and radiologic investigations, facilitates timely diagnosis and tailored management, potentially reducing associated morbidity and mortality.

## AUTHOR CONTRIBUTIONS

**Shritik Devkota:** Conceptualization; investigation; project administration; supervision; visualization; writing – original draft; writing – review and editing. **Samiksha Lamichhane:** Conceptualization; investigation; project administration; supervision; visualization; writing – original draft; writing – review and editing. **Saurabh Baghi:** Supervision; visualization; writing – original draft; writing – review and editing. **K. C. Suraj:** Investigation; methodology; writing – original draft; writing – review and editing. **Harsha Bhola:** Supervision; writing – original draft; writing – review and editing.

## FUNDING INFORMATION

The authors declare that they have no known competing financial interests or personal relationships that could have appeared to influence the work reported in this paper.

## CONFLICT OF INTEREST STATEMENT

The authors have declared that no competing interests exist.

## ETHICS STATEMENT

The authors declare that the procedures were followed according to the regulations established by Clinical Research and Ethics Committee and to the Helsinki Declaration of the World Medical Association updated in 2013.

## CONSENT

Written informed consent was obtained from the patient to publish this report in accordance with the journal's patient consent policy.

## Data Availability

Data sharing is not applicable since no new data were generated or analyzed.
